# The Case for a Quantitative Approach to the Study of Nonnative Accent Features

**DOI:** 10.1177/00238309241256653

**Published:** 2024-08-07

**Authors:** Mónica Anna Wagner, Mirjam Broersma, James M. McQueen, Roeland van Hout, Kristin Lemhöfer

**Affiliations:** Donders Centre for Cognition, Donders Institute for Brain, Cognition, and Behaviour, Radboud Universiteit, The Netherlands; Centre for Language Studies, Radboud Universiteit, The Netherlands; Donders Institute for Brain, Cognition, and Behaviour, Radboud Universiteit, The Netherlands; Centre for Language Studies, Radboud Universiteit, The Netherlands; Donders Institute for Brain, Cognition, and Behaviour, Radboud Universiteit, The Netherlands

**Keywords:** Foreign accent, accent features, data-driven approaches, Dutch-accented English, hierarchy of errors

## Abstract

Research with nonnative speech spans many different linguistic branches and topics. Most studies include one or a few well-known features of a particular accent. However, due to a lack of empirical studies, little is known about how common these features are among nonnative speakers or how uncommon they are among native speakers. Moreover, it remains to be seen whether findings from such studies generalize to lesser-known features. Here, we demonstrate a quantitative approach to study nonnative accent features using Dutch-accented English as an example. By analyzing the phonetic distances between transcriptions of speech samples, this approach can identify the features that best distinguish nonnative from native speech. In addition, we describe a method to test hypotheses about accent features by checking whether the prevalence of the features overall varies between native and nonnative speakers. Furthermore, we include English speakers from the United States and United Kingdom and native Dutch speakers from Belgium and The Netherlands to address the issue of regional accent variability in both the native and target language. We discuss the results concerning three observed features. Overall, the results provide empirical support for some well-known features of Dutch-accented English, but suggest that others may be infrequent among nonnatives or in fact frequent among natives. In addition, the findings reveal potentially new accent features, and factors that may modulate the expression of known features. Our study demonstrates a fruitful approach to study nonnative accent features that has the potential to expand our understanding of the phenomenon of accent.

## 1 Introduction

It is usually fairly evident when someone speaking in your native language has another language as their mother tongue—their speech often bears the traces of a so-called “foreign” or nonnative accent. In a classic study of accent detection, Flege (1984) even found that native listeners were able to reliably distinguish nonnative speakers from native ones with as little as the 30 ms of speech corresponding to a stop burst. Despite this apparent ease with which listeners are able to detect nonnative speech, there have been very few systematic studies addressing what features actually constitute a nonnative accent. Here, we describe an approach to examine features of nonnative accents.

Research with nonnative speech spans many different subfields and topics beyond accent recognition (e.g., Scovel, 2014) such as second-language sound learning (e.g., Reiterer et al., 2011) and the development of automatic speech recognition technology (e.g., Witt, 2012). Most empirical studies with nonnative speech concentrate on one or a few well-documented features of the accent at hand (e.g., Drozdova et al., 2016; Wagner et al., 2021; Weber et al., 2011, 2014). These features can be detected by what are often referred to as “shibboleths” (Prokić et al., 2012), following a biblical story in which shibboleth was used as a code word to detect people from a certain group who were known to have difficulty pronouncing the initial [ʃ] sound. Here, we will refer to the nonnative realizations that result from such shibboleths as “features” of the nonnative accent, not to be confused with the basic phonological features that designate certain articulatory properties of sounds, such as voicing or nasality.

### 1.1 Review of existing approaches

While studies on specific features have taught us a lot about nonnative speech processing such as the role of perception in sound production (e.g., Broersma et al., 2010; Thorin et al., 2018), it remains to be seen whether such findings generalize to other, lesser-known features of an accent. For this, extensive studies providing an overview of the features of a nonnative accent are invaluable. In this study, we aim to demonstrate the value of such extensive analyses using the English pronunciation of native speakers of Dutch—Dutch-accented English—as an example. Information about the prevalence of accent features and the potential discovery of new ones could license new insights into the mechanisms of nonnative production itself and into how such features are perceived and utilized during speech perception.

Thanks to substantial work in the field of pronunciation training, several detailed overviews of nonnative accents exist (e.g., for Dutch-accented English: Jenner, 1987; Smakman, 2014, 2019; Tops et al., 2001). Some of these even provide “hierarchies of errors” to be avoided by nonnative speakers because they may lead to misunderstandings, cause “distraction, irritation or amusement” in natives (Collins & Mees, 2003, p. 290), or simply can be easily detected. Despite their utility, it has been noted that most pronunciation guides have been developed on the basis of observations and impressions, and so far little empirical work exists to support them (e.g., Cucchiarini et al., 2012, 2014; van den Doel, 2006; van den Doel & Rupp, 2014). As a result, there is little data to bolster the proposed features, as well as to indicate how common they are in the nonnative sample, or how uncommon they are among native speakers. Furthermore, since these overviews were originally intended for use during pronunciation instruction, they tend to prioritize features that may affect a speaker’s intelligibility or signal nonnative status. As such, they may exclude features that are less obvious or cumbersome for comprehension but nonetheless deviate from native realizations. Moreover, because they are subjective, pronunciation guides and their error hierarchies may vary based on what their creators choose to prioritize (e.g., detection, intelligibility, stigmatization). The collection and systematic analysis of data on feature occurrence could provide an objective quantification of the differences between natives and nonnatives, as well as the relative prevalence of the features among both groups. Moreover, it could signal features that are lesser-known—perhaps because they are less salient or prevalent—or even lead to the discovery of previously undocumented features (Bloem et al., 2016). This information would be useful, for example, for language research or even pronunciation training, providing an objective, quantitative foundation from which to work.

Of the few extensive experimental studies of nonnative accent features that do exist, many make use of ratings of nonnative speech. For example, van den Doel (2006) had native British and American English speakers judge the severity of a list of pre-selected errors that Dutch speakers tend to make, resulting in two hierarchies of error. However, as the differences between the two hierarchies can attest to, factors such as the raters’ native status, phonetic expertise, or native variety can all influence the features indicated and their relative importance (Koet, 2007). Moreover, many empirical studies, such as this one, still employ some form of pre-selection of the features they address which, while informative—for example, regarding the salience, persistence, and stigmatization of certain features—does not provide more specific information about the pronunciation errors that actually occur and their frequency (Cucchiarini et al., 2014).

Cucchiarini and colleagues (2014), in their study of Dutch-accented English, used both a top-down, knowledge-based approach by selecting nonnative features based on the literature and language teacher impressions, and a bottom-up, data-driven approach, where they let the features emerge themselves from the analysis of nonnative speech samples. They did this by having the speech phonetically transcribed and calculating the proportion of times each sound deviated from the standard British pronunciation, or an accepted variant, of each word. As a result, they created a ranking of the most frequently mispronounced sounds, indicating their most frequent substitutions. Although the bottom-up approach confirmed many of the features expected based on the observational studies and teachers’ impressions, some differences existed.

Cucchiarini and collaborators’ (2014) approach of using a native standard against which to compare the pronunciation of nonnatives is common in research into nonnative accent features. However, while using a standard accent of a language is useful when it comes to teaching the language, it is undeniable that native speakers themselves vary quite a bit from those standards. Moreover, nonnatives may differ in the standards they aim for (e.g., Received Pronunciation, RP, or General American, GA, for English). Thus, by comparing nonnative realizations to an English standard, a feature may be considered nonnative while it is actually a feature of another, non-standard variety. Moreover, much remains to be understood about how native speakers perceive standard and non-standard features, with evidence that they may even rate some features present in their own non-standard variety as nonnative-like (van den Doel, 2006). Gimson and Cruttenden (1994, cited in van den Doel, 2006) wrote that “it is wiser to listen to the way in which the native speaks rather than ask his opinion” (pp. 196–197). To really be able to say what is native-like or not, one must find where the pronunciation of a nonnative speaker varies from different English accents. This is possible with a method proposed and demonstrated by Bloem et al. (2016), developed in the field of dialectometry (Nerbonne et al., 2011; Prokić et al., 2012), in which the pronunciation of nonnative speakers, transcribed, is compared with the actual pronunciation of native speakers, also transcribed. With this method, a score is automatically calculated for each item (in their case: word) based on how differently it is realized by two groups of speakers (e.g., native and nonnative speakers of English). Moreover, in their implementation, which makes use of Levenshtein distances, it is possible to, to a certain degree, take into account phonetic distance: realizations varying only in diacritics receive smaller distances than realizations in which the phonemes differ.

Bloem and colleagues (2016) applied their analysis at the word level, yielding rankings of the words from the elicitation material that were most characteristic of the nonnative accents included. In this study, we demonstrate how applying this method at the sound-level can provide more detailed information about the distinctive features of the accent at hand. However, one characteristic of this method and of data-driven approaches in general is that the underlying features need to be deduced, that is, while the analysis can give an indication of how differently certain sounds are pronounced by natives and nonnatives, determining the source of that difference is still up to the researcher. Here, in a second step, we demonstrate a method to test hypotheses about underlying accent features which consists in first determining the relative prevalence of the proposed feature (i.e., its frequency relative to the opportunities for it to occur in the elicitation material; as Cucchiarini et al., 2014] did). Next, these relative frequencies are statistically analyzed to test whether the native and nonnative groups vary in the degree to which they express each feature overall.

In addition to aiming for different native standards, nonnative speakers’ regional accents may also influence their pronunciation in a second language (Simon et al., 2015; Tops et al., 2001). While some studies try to take into account well-known features of certain regional varieties (e.g., Collins & Mees, 1993, 2003; Gussenhoven & Broeders, 1997; Koet, 2007; van den Doel, 2006), few examine this with data, where less well-known or salient but nonetheless distinctive features can arise. To do this, a sample should include speakers from different regions. Furthermore, in addition to regional accents, when it comes to a second language, proficiency can play a large role in how speakers are found to deviate from native pronunciation and thus in the accent features in question (Cucchiarini et al., 2014). Moreover, each speaker has their own idiosyncratic way of speaking. The method recommended by Bloem and collaborators (2016) takes into account this variability for both nonnative and native speech by aggregating over data from many speakers. However, our additional statistical method can also be used between groups to test hypotheses about differences between regional accents. We demonstrate this here by comparing the English pronunciation of two nonnative groups (i.e., Dutch speakers from Belgium and The Netherlands) to two native groups (i.e., English speakers from the United States and U.K.) to see if they deviate from native English speakers in different ways. Finally, other factors such as the target sound or its position in a word or syllable can affect the degree to which certain features are expressed (Cucchiarini et al., 2014). The statistical approach can also be applied within a feature to test the incidence of additional factors, potentially shedding further light on nonnative speech processing.

### 1.2 Present study

Despite the abundance of research on nonnative speech, there is a marked lack of extensive, objective analyses of the features that actually distinguish nonnative speech from that of natives. In this study, we present a twofold quantitative approach to determine features of a nonnative accent. By way of illustration, we will describe the application of this approach to the analysis of Dutch-accented English and discuss a selection of results thereof. We first describe the application of a dialectometric method (Bloem et al., 2016) which can be used to aggregate over phonetic distances between native and nonnative realizations to provide a measure of how distinctive of Dutch-accented English each item is. Examining the patterns of realizations by the native and nonnative speakers can provide an indication of the underlying accent features of the most distinctive items.

Next, we describe a statistical approach which can be used to verify hypotheses about accent features which consists of testing whether the prevalence of the features overall varies between the native and nonnative speakers. Furthermore, we demonstrate how this method can be used to address regional differences in the nonnative speakers’ native language, as well as in the target language, by analyzing native speakers of both Netherlands and Flemish Dutch and in comparison with native speakers from both the United States and United Kingdom. We thus compare the features of Dutch-accented English of speakers from the Netherlands and from Belgium in relation to both groups of native speakers. By aggregating over many speakers, this method also takes into account speaker variability. In addition, we demonstrate how this approach can be used to examine whether features are differentially expressed in varying conditions (e.g., for different sounds or in different positions or phonetic contexts). We are not aware of many other studies that try to provide an extensive overview of the features of a nonnative accent in an empirical way that also takes into account speaker variability. By comparing our findings to existing error hierarchies obtained with other methods, we hope to demonstrate the value of this and other quantitative approaches for the study of nonnative accent features.

## 2 Methods

### 2.1 Materials

The material for this study, as in Bloem and collaborators (2016), came from the Speech Accent Archive (SAA, http://accent.gmu.edu; Weinberger, 2015), a corpus of native and nonnative speakers reading the following elicitation paragraph out loud in English:

Please call Stella. Ask her to bring these things with her from the store: six spoons of fresh snow peas, five thick slabs of blue cheese, and maybe a snack for her brother Bob. We also need a small plastic snake and a big toy frog for the kids. She can scoop these things into three red bags, and we will go meet her Wednesday at the train station.

Crucially, for each speaker, an audio and narrow phonetic transcription is provided. The transcriptions were carried out in the International Phonetic Alphabet (IPA) by three phonetically trained transcribers, with each transcription being agreed upon by all three transcribers (Weinberger & Kunath, 2011). A more recent reliability study (cited in Gao & Weinberger, 2018), in which 67 additional transcribers performed transcriptions of samples from the archive, found that 72% of these transcriptions agreed with the original ones.

The “Stella paragraph,” as it is often referred to, was designed for use with nonnative speakers of English precisely to identify phonological speech patterns specific to different nonnative accents. It thus includes almost all of the sounds of the English language and many consonant clusters known to be difficult for L2 learners (Weinberger & Kunath, 2011). The relative frequency of each sound in the Stella paragraph also roughly corresponds to that of natural speech as reported by Edwards (1992; Weinberger & Kunath, 2011).

Although speech produced by reading out loud is unnatural and, some may argue, less ideal for judging pronunciation than spontaneous speech, it has the benefit of allowing for a more controlled comparison of speakers’ pronunciation. Moreover, there is evidence that read and unread speech may not be rated differently in terms of accentedness (e.g., Munro & Derwing, 1994; Scheuer, 2002).

The sample for the present study consisted of all of the speakers for whom transcriptions were available in the archive and who were born in Belgium (BE, *n* = 25), the Netherlands (NL, *n* = 16), the United States (*n* = 121), or the United Kingdom (UK, *n* = 22). Demographic information about the speakers and their birthplaces can be found in the supplementary material available in the Radboud Data Repository (https://doi.org/10.34973/bpg8-ff82).

The transcriptions were made available in text files by Steven Weinberger. We manually aligned (on a word level) and segmented them. In their study, Bloem and collaborators (2016) performed their statistical analyses on the Stella paragraph on a word level, thus obtaining a ranking of the words in terms of how characteristic their pronunciation was of Dutch speakers. However, as it is impossible to determine with certainty which phonetic segment is responsible for the word’s ranking, word-level results are not very generalizable. Here, we carried out the alignments on the segmental level (i.e., |*p*|l|iː|*z*| for “please”), with each slot designating an “item” here. Unless otherwise specified, we based our alignments on the words’ citation forms (e.g., |æ|*n*|d| for “and”) rather than how the word can be pronounced when not stressed (e.g., |ə|n| or |n|). This also meant that for some realizations, consecutive segments were considered one item to ensure they were compared properly. For example, “things” produced as [θɪŋɡz] and [θɪŋks], instead of the standard [θɪŋz], were segmented as: |θ|ɪ|ŋɡ|*z*| and|θ|ɪ|ŋk|s|, to ensure the distance between [ŋɡ] and [ŋk] relative [ŋ] was calculated. Similarly, “her” produced as [həɹ], instead of [hɜː] or [hɚ], was segmented into two items: |h|əɹ| to ensure alignment of the rhyme.

For our segmentations, we took into account the English standard pronunciations RP and GA. For example, the word “store” was segmented into three parts: one for the initial consonant [s], one for the middle consonant [t], and a third for the rhyme, because, depending on the standard, this could consist in one or two segments: [ɔː] or [ɔːɹ]. To use the same segmentation for all accent groups, we opted to consider multiple sound segments (e.g., [ɚː] and [ɹ]) as one item when the standards were in discordance. This yielded a total of 219 items for analysis, which can be found in Appendix A. Where necessary, the transcriptions were corrected to omit speech disfluencies and include missing spaces.

### 2.2 Measure to identify distinctive accent features

Once aligned and segmented, the transcriptions were uploaded to Gabmap (https://gabmap.let.rug.nl/; Nerbonne et al., 2011), a web-based application for dialectometry. Gabmap handles matrices of locations (dialects) by linguistic items (commonly words, as in Bloem et al., 2016). In our case, the locations are our speakers and the linguistic items are the sound items. Gabmap can calculate three measures developed by Nerbonne and colleagues (e.g., Prokić et al., 2012; Wieling & Nerbonne, 2011): representativeness, distinctiveness, and characteristicness. The distinctiveness measure yielded by Gabmap, referred to there as “between” (group) differences or scores (Leinonen et al., 2016), is a standardized form of the mean Levenshtein distance between the nonnative speakers and native speakers so that items ranked high in distinctiveness are items where the differences between nonnatives and natives are large (Bloem et al., 2016; Wieling & Nerbonne, 2011). Bloem and colleagues (2016), in their word-level analysis of the Stella paragraph, focused on the characteristicness scores. However, this measure favors items where the nonnatives agree on their realization of the nonnative-like deviation over items where nonnatives may deviate from the natives to the same degree, but present more variability in their exact realization. Since we are interested in which sounds Dutch speakers produce differently from natives, regardless of whether they tend to agree on their exact realization of the sounds in that deviation, we will make use of the distinctiveness scores here. The formula used by Gabmap to calculate distinctiveness, with the variables adapted for our terminology, is

$${\bar{d}}_{i}^{\neg l}=\frac{1}{\left|l|(|G|-|l|\right)}\sum_{s\in l,s^{\prime }\notin l}d_{i}(s,s^{\prime })$$

or the mean difference *d̅* with respect to a particular item *i* from the group of native speakers of a language *l* (here: Dutch; expressed as the difference from speakers not in that language group or *¬l*, here: native English speakers) is equal to the sum of Levenshtein distances *d* for that item *i* between the realizations by any speaker *s* in the native language group *l* and another speaker *s’* not in the native language group, divided by the number of pairwise comparisons between speakers. The number of comparisons can in turn be calculated by multiplying the number of native speakers of the language, |*l*|, with the total number of speakers, |*G*|, of all the languages considered (here: Dutch and English) minus the number of native speakers of the language, |*l*|.

This measure is then normalized for comparability across items by subtracting the mean Levenshtein difference between all pairs of speakers (both native and nonnative) for that item, *d̅_i_*, and dividing by the standard deviation rendering a distinctiveness score, much like a z-score:

$$\frac{{\bar{d}}_{i}^{\neg l}-{\bar{d}}_{i}}{sd(d_{i})}$$

Gabmap employs an adaptation, described in Nerbonne and Heeringa (2010), of Levenshtein distances, a measure of the number of simple operations (insertions, deletions, and substitutions) required to transform one string into another. For example, according to this adaptation, the distance between [z] and [z] is 0 because no changes are necessary, between [z] and [z̥] it is 0.5 because there is only a difference in a diacritic, and between [z] and [s] it is 1 because an entire segment would need to be changed. These adapted distances, which aim to approximate phonetic distances, have been found to correlate well with accentedness ratings (Wieling et al., 2014).

We uploaded the transcriptions of the Stella recordings for each combination of nonnative-native groups: NL-US, NL-UK, BE-US, and BE-UK. Gabmap returned the three types of scores (representativeness, distinctiveness, and characteristicness) for each item in the paragraph, which allowed us to create a ranking of items from the most to the least distinctive of that variety of Dutch-accented English compared with native speakers of the target regional variety.

We then analyzed the most distinctive items of each of the four rankings and the most common native and nonnative realizations for each item to extract the underlying nonnative feature. To determine the features underlying the deviations observed, we also consulted previous literature on features of Dutch-accented English and Dutch itself. As distinctiveness is represented as a continuum: the higher the score, the more distinctive the item, we decided to use a cut-off and only inspect the first 25 (equivalent to the top 11.4%) items of each ranking. Table 1 shows an excerpt of the top five items in the NL-UK comparison ranked by Gabmap’s distinctiveness score, listed along with the word-context of each item, both the natives’ and nonnatives’ most frequent realization of the item, and the nonnative accent feature we think underlies each item. Please note that the names of the features are based on how the nonnative speakers varied from the natives, and therefore often designate the absence of a feature (e.g., nonnatives fail to aspirate or to use the weak form). From the top 25 items of the four rankings, 14 unique features were observed (presented in Table 2).

**Table 1.** Top 5 of 25 Items Analyzed in the UK-NL Comparison.

Rank	Distinctiveness score	Item		Most frequent realizations		Feature
		#	in context	UK	NL	
1	0.61	76	slabs	z	s	word-final obstruent devoicing
2	0.59	149	the [2]	ð	d	dental fricative substitution
3	0.58	1	please	pʰ	p	initial voiceless plosive deaspiration
4	0.57	68	five	v/v̥	f	word-final obstruent devoicing
5	0.53	65	peas	z/z̥	s	word-final obstruent devoicing

*Note*. The item corresponds to the underlined part of the word, with the # designating the item number (index). The most frequent realizations correspond to realizations with relative frequencies ⩾ 25%.

**Table 2.** The Features Extracted From the Top 25 of All Four Pairs, With Examples of Frequent Nonnative Realizations and the Comparisons in Which They Were Observed (Indicated With ✓).

Feature	Example^a^	NL-US	NL-UK	BE-US	BE-UK
word-final obstruent devoicing	please	✓	✓	✓	✓
	[z] → [zɾ]				
dental fricative substitution	the	✓	✓	✓	✓
	[ð] → [d]				
initial voiceless plosive deaspiration	call	✓	✓	✓	✓
	[kʰ] → [k]				
/æ/ substitution	ask	✓	-	✓	✓
	[æ] → [a]				
alveolar stop unflapping	to	✓	-	✓	-
	[ɾ] → [t]				
weak form unweakening	to	-	✓	-	✓
	[ə] → [u]				
regressive voice assimilation	scoop these	✓	-	-	-
	[p] → [b]				
/ɪ-i/ conflation	thick	-	-	✓	-
	[ɪ] → [i]				
approximant devoicing	slabs	-	-	✓	-
	[l] → [l̥]				
alveolar trill *r*	bring	-	-	✓	-
	[ɹ] → [r]				
dark *l* palatalization	call	-	-	-	✓
	[ɫ] → [l]				
/eɪ/ diphthongization	Wednesday	-	-	-	✓
	[e] → [eɪ]				
open back vowel unrounding	Bob	-	-	-	✓
	[ɒ] → [ɑ]				
/i:/ monophthongization	peas	-	-	-	✓
	[ə̆ɪ] → [i]				

a Context and example of common native realization → common nonnative realization.

### 2.3 Analysis of feature prevalence

Because distinctiveness is calculated on an item level, each feature can appear multiple times in the resulting rankings. However, features that appear frequently are not necessarily more distinctive than others; rather they may simply have had more opportunities to occur, which could be a consequence of the paragraph’s construction or of the nature of the feature itself (e.g., words ending in voiced obstruents occur more often in English than does the voiced labio-velar approximant /w/). The ranking itself thus does not provide an indication of the feature’s prevalence but rather of that of the item in particular (i.e., of that sound in that word and context). To verify that the features designated for the most distinctive items are themselves distinctive, we examined the frequency with which they occurred. For this, we took into account all items in the passage where each feature could potentially have appeared (e.g., all words ending in a voiced obstruent for final devoicing; a similar relative frequency approach was adopted per sound in Cucchiarini et al., 2014; Koster & Koet, 1993). The items considered for each of the features discussed here are indicated in Appendix B. For all the items of a feature, each realization was then classified in terms of whether the feature had occurred or not (1 = feature present, 0 = feature absent). This was done for all of the native and nonnative speakers. Thus, this also enabled us to determine the prevalence of each feature per group of speakers and to check whether the feature appeared more often in the speech of the Dutch speakers than the native English speakers. Null realizations were considered missing values in the data, except when meaningful, as in omission of the “h” in “her” which counted as producing the weak form.

As already mentioned in the Introduction, some features may only appear in certain conditions or else they may occur to varying degrees in different conditions. For example, final devoicing may be differentially expressed for different sounds. Likewise, the expression of other features may be influenced by the position in the syllable or word of the item in question or by its surrounding context. When we suspected this could be the case for a feature and enough instances were available, we also tested whether these conditions had a significant influence on the feature’s presence.

We analyzed the data with generalized linear mixed-effects modeling (GLMM) for binary outcomes in *R* (version 4.0.2.; R Core Team, 2020) using the glmer function from the lme4 package (version 1.1-23; Bates et al., 2015) and a logit link function following the procedure outlined in Bolker and collaborators (2009). For each feature, we tried to fit a model that would predict the probability of a feature being expressed based on the speaker group (BE, NL, UK, US) and, if applicable, condition (with the levels corresponding to different sounds, contexts, or positions). Mixed-effects modeling was used to account for the fact that the observations were not independent but rather each speaker provided multiple realizations and each item was produced by all speakers (Gries, 2015; Jaeger, 2008). We followed a maximal modeling approach (Barr et al., 2013), first trying to find a maximal random-effects structure that was supported by the data (Brauer & Curtin, 2018; Gries, 2015, 2021). The inclusion of random intercepts for speaker and item was confirmed with log-likelihood significance tests (Baayen, 2008; Zuur et al., 2009). Random slopes for speaker group and, if applicable, condition were also tested but usually resulted in singularity errors and thus had to be excluded. Once the random-effects structure was established, the inclusion of a fixed effect for speaker group and, if applicable, for condition, as well as an interaction between the two, was tested. Inclusion of each fixed effect was considered justified when decreased Akaike Information Criterion (AIC) values indicated that it significantly improved model fit compared with a model without it (Baayen et al., 2008; Gries, 2015; Jaeger, 2008). When a factor was found to improve the model, we did post hoc pairwise comparisons between the estimated means of the different levels of the factors using emmeans (version 1.7.0; Lenth, 2021), given the unbalanced nature of the data. However, caution should be exercised when interpreting these results when the factors were found to interact. P values were corrected for multiple comparisons using the False Discovery Rate (FDR). Unless otherwise noted, model goodness-of-fit was determined to be adequate for all models with posterior predictive checks (Bates et al., 2015; Gelman et al., 1996, 2000).

## 3 Results and discussion

The 25 most distinctive items for each of the four comparisons, as determined by the analysis in Gabmap, can be found in Appendix C, but Table 2 provides an overview of the 14 features extracted from the items and the rankings atop which they appeared.

As you can see from the table, some features appeared at the top of all four rankings, while others were only featured in select native-nonnative comparisons. In what follows, we will discuss our results concerning three features, two of which are well-known and one which is potentially new. We have chosen to discuss these features in particular because we think they highlight what this approach can contribute, even to the knowledge about well-known accent features. We will start our discussion of each feature with a short description of the feature in general and how it was realized by the speakers in the archive, followed by the results of our statistical analyses on each feature’s prevalence among the four speaker groups. We will briefly compare our findings to those of other studies. Finally, we will discuss “unobserved” features, which were not present in our top rankings despite commonly appearing in descriptions of Dutch-accented English.

A summary of the appearance of the features we observed in other error hierarchies can be found in Table 3. In addition, a table summarizing each of these hierarchies can be found in the supplementary material available in the Radboud Data Repository (https://doi.org/10.34973/bpg8-ff82).

**Table 3.** An Overview of the Presence (✓) and Absence (-) of the Most Distinctive Features Found Here in Other Dutch-English Error Hierarchies.

Feature	Collins et al. (2018)	Cucchiarini et al. (2014): bottom-up	Cucchiarini et al. (2014): top-down	van Hattum & Rupp (2014)	Hoorn et al. (2014)	Rupp (2013)	van den Doel (2006)	Collins & Mees (2003)	Tops et al. (2001)	Collins & Vandenbergen (1998)	Gussenhoven & Broeders (1997)	Collins & Mees (1993)	Koster & Koet (1993)
word-final obstruent devoicing	✓	✓	✓	✓	✓	✓	✓	✓	✓	✓	✓	✓	-
dental fricative substitution	✓	✓	✓	✓	✓	✓	✓	✓	✓	✓	✓	✓	✓
initial voiceless plosive deaspiration	✓	-	✓	✓	✓	✓	✓	✓	✓	✓	✓	✓	-
/æ/ substitution	✓	✓	✓	✓	✓	✓	✓	✓	✓	✓	✓	✓	✓
alveolar stop unflapping	-	-	-	-	-	-	-	-	-	-	-	-	-
weak form unweakening	✓	✓	-	-	✓	✓	✓	✓	✓	✓	✓	✓	-
regressive voice assimilation	✓	-	-	-	-	✓	-	✓	-	✓	✓	✓	-
/ɪ-i/ conflation^a^	-	✓	-	-	-	-	-	✓	✓	✓	-	✓	-
approximant devoicing	-	-	-	-	-	-	-	-	-	-	-	-	-
alveolar trill *r*	-	-	-	-	-	✓	-	-	-	-	✓	-	-
dark *l* palatalization	-	-	-	-	-	-	-	-	-	-	-	-	-
/eɪ/ diphthongization	-	-	-	-	-	-	-	-	-	-	-	-	-
open back vowel unrounding	-	✓	-	-	-	-	-	-	-	-	✓	-	-
/iː/ monophthongization	-	-	-	-	-	-	-	-	-	-	-	-	-

a Note that these studies only mention a more close realization of /ɪ/ with no reference to prevelar raising, suspected here.

### 3.1 Observed features

#### 3.1.1 Dental fricative substitution

A feature present in all four speaker group comparisons concerns the realization of dental fricatives (voiced /ð/ and voiceless /θ/). These are sounds that many nonnative speakers of English have difficulty with as many languages, including Dutch, lack these fricatives in their native inventories.

**Figure 1.**
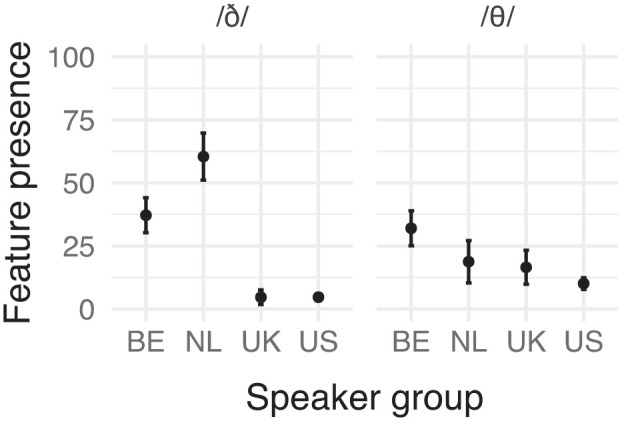
Relative frequency (% of realizations observed) of dental fricative substitutions per sound and speaker group. Error bars represent 95% confidence intervals.

This feature is also well-known and appeared in all other error hierarchies consulted. In fact, Koster and Koet (1993) reported that more than 50% of the consonant pronunciation errors detected by native English speakers in their study concerned a dental fricative. However, some studies only mention the voiced fricative /ð/. This may be because the voiceless dental fricative /θ/ is often considered easier for nonnative speakers to produce than the voiced version (Collins & Mees, 2003; Smakman & de France, 2012). That perhaps explains why /θ/ only appeared at the top of one of our rankings—that comparing the BE and US speakers. A summary of the feature’s prevalence in our data is shown in Figure 1, where you can find the number of times non-target-like realizations were observed relative to the number of opportunities for them to occur in the passage (i.e., occurrences of dental fricatives). We report the feature’s prevalence for each speaker group and dental fricative in question, which we refer to as the factor “sound.” The mean proportions of feature presence for this and the other two features discussed can be found in Appendix D.

In Figure 1 you can see that the BE and US groups showed the highest and lowest rates of this feature for /θ/, respectively. Furthermore, our results suggest that only the NL speakers seem to have produced more non-target realizations for /ð/ than /θ/.

The results of GLMM indicated that a model including an interaction between speaker group and sound best fit the data and improved model fit over a model without the interaction, χ^2^(3) = 42.938, *p* < .001.^1^ Table 4 shows the pairwise comparisons between speaker groups per sound (for comparisons between sounds, see Appendix E). The estimated marginal means on the diagonals (gray background) represent the estimated probability of the feature appearing for each sound and speaker group combination. In the remainder of the cells, we report the results of the paired comparisons: the cells forming a triangle below the diagonal contain the comparisons between the estimated marginal means of the two sounds or speaker groups while the significance of these comparisons (*p* values) can be found in the triangle of cells above the diagonal. The comparisons between speaker group-sound combinations are expressed as an odds ratio, for example: dental fricative substitution of the sound /ð/ is 33.883 times as likely to be substituted among speakers from Belgium than among speakers from the United Kingdom.

**Table 4.** Estimated Marginal Means (Probability, Diagonal) of Dental Fricative Substitution Per Speaker Group and Comparison Thereof for Each Sound (Odds Ratio: Lower Triangle, and Significance: Upper Triangle).

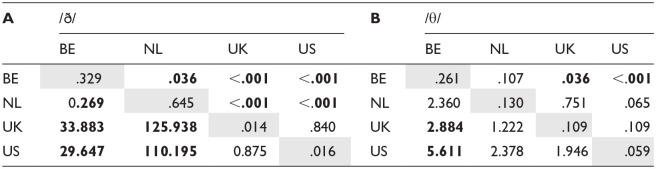

*Note*. Diagonal (gray cells): estimated marginal means (estimated probability of feature presence); lower triangle: comparison of means (odds ratio); upper triangle: significance of comparisons with FDR adjustment. Significant *p* values (<.05) are displayed in bold. Comparison of estimated marginal means between sounds per speaker group can be found in Appendix E (Table 4, C-F).

As can be seen from Figure 1 as well as the results of the pairwise comparisons in Table 4, while there is a large difference between the Dutch and English groups for the voiced /ð/, for the voiceless /θ/ only the BE speakers varied from the natives (although there is a trend for the NL-US comparison).

The disparity between the voiced and voiceless fricatives is echoed in the literature: Koster and Koet (1993) found that /ð/ constituted 47.3% of the pronunciation errors detected by native English speakers, while only 7.8% were for /θ/. Similarly, only /ð/, but not /θ/, appeared among Cucchiarini and collaborators’ (2014) bottom-up list of most frequent mispronunciations. However, our results suggest that this may only hold for speakers from The Netherlands and perhaps reflects a bias in the literature.

Despite dental fricative substitution being a well-known feature of Dutch-accented English, Bloem et al. (2016) noted in their demonstration with Gabmap that words with “th” only appeared at the top of the chart when the ranking was by distinctiveness scores, which only contemplates deviation from the natives. Conversely, these words appeared low on the ranking when it was by characteristicness, which takes into account whether the nonnatives agreed in their realizations, which they attributed to the diversity with which Dutch speakers realize the dental fricatives.

This is true for the voiceless fricative /θ/, which is said to be substituted by [t, s] and sometimes [f] (Collins et al., 2018; Collins & Mees, 1993, 2003; Collins & Vandenbergen, 1998; Cucchiarini et al., 2014; Gussenhoven & Broeders, 1997; Rupp, 2013). However, while Dutch speakers have been found to replace /ð/ with [d, z, t, s, θ, f] (Collins et al., 2018; Collins & Mees, 1993; Collins & Vandenbergen, 1998; Cucchiarini et al., 2014; Gussenhoven & Broeders, 1997; Rupp, 2013), most studies agree that [d] is most common, accounting for more than 90% of the pronunciation errors for /ð/ reported by Cucchiarini and collaborators (2014).

Our results are in line with the literature, with by far the most frequent non-native replacements for /ð/ being [d] and [d̪] (dental /d/) and for /θ/: [t̪] (dental /t/), [t, s]. In addition, Collins and Mees (2003, p. 142) put forth that Netherlands Dutch speakers tend to replace /θ/ with [s] more than speakers from Belgium. Our realization data seem to follow this pattern, although not tested systematically. It is worth noting, however, that the particular sound substituted is thought to be linked to the position in the syllable where the dental fricative occurs (Collins & Mees, 1993; Cucchiarini et al., 2014; Gussenhoven & Broeders, 1997). In our data, nearly all tokens of the two fricatives, except for one item each, occurred at word-onset.

In summary, our data supports the idea that Dutch speakers produce non-target realizations of the dental fricatives. Moreover, our results suggest a difference between /ð/ and /θ/ at least for NL, with substantially more replacements for the voiceless fricative. Finally, the substitutions in particular we observed for these sounds were generally in line with past findings.

#### 3.1.2 /æ/ substitution

The feature of Dutch-accented English that has probably received the most attention in the scientific literature concerns the pronunciation of the English phoneme /æ/ (e.g., Broersma et al., 2010; Thorin et al., 2018). This sound does not exist in the native Dutch inventory and research suggests that Dutch speakers often realize it like the English /ɛ/, which is closer to the Dutch /ɛ/, rendering words such as “bat” more like “bet” (e.g., Wang & van Heuven, 2006). This phenomenon is often referred to as /æ/~/ɛ/ conflation.

The sound /æ/ is the vowel that occurred most often in our rankings, appearing at the top of all comparisons except that of NL-UK, where it first appeared in position 84. In our frequency analysis, we considered all variants that were not [æ] nor [æː] as presenting this feature. Here, it is relevant to keep in mind that there is a set of words, known as the “BATH words,” that most speakers from the United States and the United Kingdom pronounce with [æ] (as in TRAP), but speakers of non-regional British English and standard southern English pronounce with [ɑː] (as in PALM; Collins & Mees, 2008). However, only one item considered for this feature (i.e., “ask”) was among this set, and the data reveal that only a minority of the UK speakers produced [ɑː] for this item.

**Figure 2.**
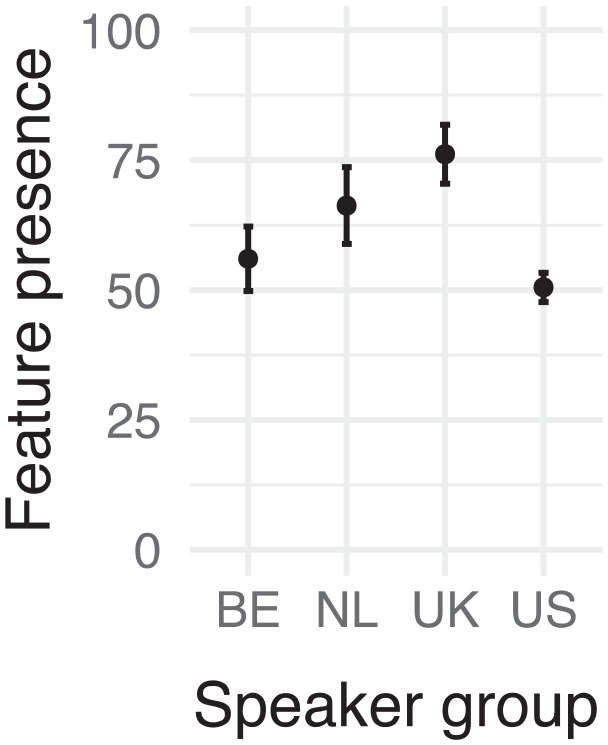
Relative frequency (% of realizations observed) of /æ/ substitution per speaker group. Error bars represent 95% confidence intervals.

The prevalence of non-target [æ] realizations in our groups, visible in Figure 2, at first glance does not seem to align with the high distinctiveness scores for this feature seen in our rankings; this feature appears to be quite frequent among native speakers.

**Figure 3.**
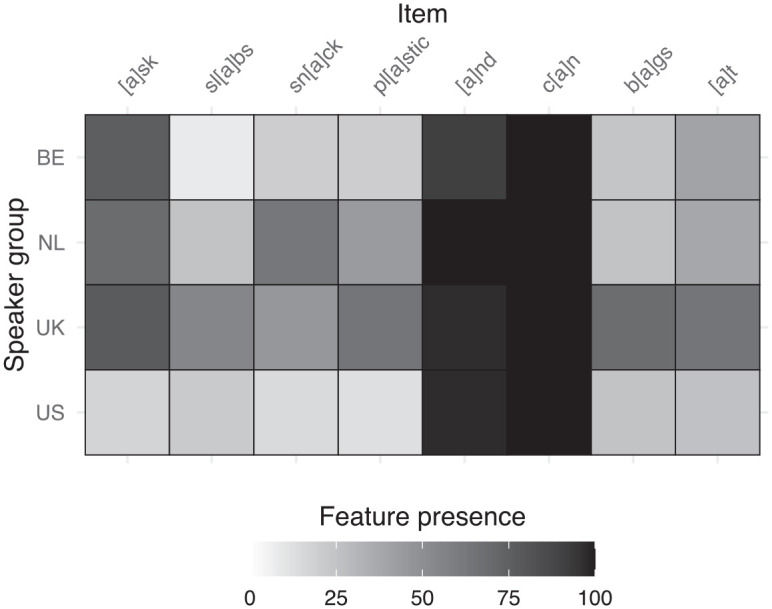
Relative frequency (% of realizations observed) of /æ/ substitution per word and speaker group.

From the plots of feature presence per word in Figure 3, we can see that almost all speakers, regardless of speaker group, produced non-[æ] variants for the words “and” and “can.” This is because they were usually produced in their weak form with vowel reduction. Therefore, we removed these two words from the analysis.

The model with speaker group was supported by a posteriori predictive checks, χ^2^(3) = 47.254, *p* < .001. Pairwise comparisons (Table 5) revealed that this was due to the speakers from the United States producing more target-[æ] realizations than both groups of nonnative speakers in addition to speakers from the United Kingdom. The speakers from the United Kingdom even replaced /æ/ significantly more often than the Belgians.

**Table 5.** Estimated Marginal Means (Probability, Diagonal) of /æ/ Substitution Per Speaker Group and Comparison Thereof (Odds Ratio: Lower Triangle, and Significance: Upper Triangle).

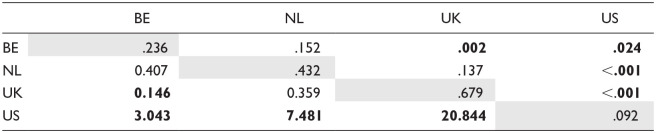

*Note*. Diagonal (gray cells): estimated marginal means (estimated probability of feature presence); lower triangle: comparison of means (odds ratio); upper triangle: significance of comparisons with FDR adjustment. Significant *p*-values (<.05) are displayed in bold.

This is not the pattern we would expect for a feature with high distinctiveness, but closer examination of the items in the top rankings supports these findings. There we can see that “ask” and “snack” only distinguished the nonnative speakers from native speakers from the United States, but not the United Kingdom. Similarly, the high distinctiveness scores for “bags” and “slabs” seem to have been due to the UK speakers, rather than the nonnatives, producing a high proportion of non-[æ] variants. Inspection of the UK speakers’ realizations reveals that this can be attributed to these speakers frequently replacing /æ/ with other variants, including [a], a feature of modern RP (Collins et al., 2018).

Overall, our findings suggest that Dutch speakers do not consistently substitute /æ/ with other variants. In fact, for “slabs,” the majority of the Dutch speakers seem to have produced target-like realizations, suggesting that other factors may influence the tendency for nonnatives to replace /æ/, for example: perhaps phonological context, word frequency, or the loan status of the word. Furthermore, it seems that the speakers from the Netherlands tend to produce more non-[æ] variants than Belgian speakers. In addition to these points, our results suggest that when nonnative speakers produce variants, it is not always [ɛ] but can be [a]. Whether this is due to imitation of modern RP or interference from the Dutch vowel /aː/ remains to be seen. Finally, our results highlight the fact that native English speakers often deviate from the standard as well, revealing a lot of variability for the target [æ].

These findings are not what we would expect based on nonnative research with /æ/ and on the other error hierarchies consulted, where the articulation of [æ] more like [ɛ] was always present. Even in Cucchiarini and colleagues’ (2014) work with high-proficiency English speakers where as little as 1% of instances were found to be erroneous, almost 50% of these were [ɛ] realizations and 20% were [ə]. Our results thus raise the question of when Dutch speakers substitute /æ/ with [ɛ] and when with [a].

#### 3.1.3 Dark l palatalization

This feature was also only present at the top of the BE-UK ranking, although it should be noted that it appeared in positions 27, 30, and 41 in the BE-US, NL-UK, and NL-US comparisons, respectively. The feature appeared in the item corresponding to the final consonant sound in the word “call,” which a majority of the native English speakers realized as [lˠ], followed by around a third who produced [l]. In contrast, [l] was by far the most frequent realization for the Dutch speakers.

The two realizations of *l* correspond to the two main allophones of the lateral approximant in English. The clear *l*, [l], is formed with the tongue slightly raised toward the palate, forming a convex shape and producing a [i]-like quality (e.g., late). For dark *l*, [ɫ], the back of the tongue is raised toward the velum (velarized *l*, [lˠ]) or pharynx wall (pharyngealized *l*, [lˤ]), giving a concave shape and producing a [ʊ]-like sound (e.g., hole; Collins & Mees, 2003; Gussenhoven & Broeders, 1997). The two variants have a complementary distribution, with clear *l* occurring before vowels and [j], and dark *l* everywhere else, that is, before consonants, pauses (word-offset), and when *l* is syllabic (Gussenhoven & Broeders, 1997). Accordingly, the *l* in the word “call” should be dark, as most of the native English speakers produced.

**Figure 4.**
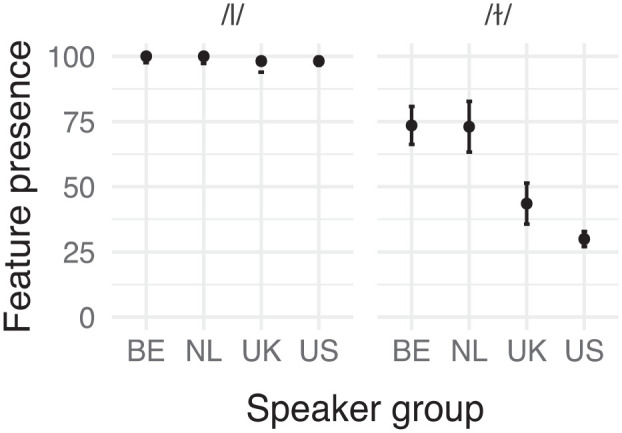
Relative frequency (% of realizations observed) of dark *l* palatalization per position and speaker group. Error bars represent 95% confidence intervals.

Standard NL and BE Dutch also have a distinction between clear and dark *l* with a similar distribution. However, many non-standard varieties only have one or the other. Based on the realization in “call,” our data seem to suggest an over-extension of the clear *l* in English, which we refer to as “dark *l* palatalization.” In Figure 4, we can see how often each group produced palatalization where clear *l* is the standard and where dark *l* is the standard. We can see that in clear-*l* positions, both native and nonnative groups overwhelmingly produced clear *l*. However, in dark-*l* positions, as in “call,” the Dutch speakers still produce palatalization at a high rate.

Due to the lack of variability in clear-*l* positions—nearly all realizations were clear *l*—, we tested for a main effect of group in the items where dark *l* is expected. The results of our statistical analysis of the feature’s prevalence indicated that speaker group significantly contributed to the model, χ^2^(3) = 40.433, *p* < .001. Pairwise comparisons, reported in Table 6, confirmed what we see in the second plot in Figure 4: where a dark *l* could occur, before consonants and pauses, the Dutch speakers produced a clear *l* much more often than the native English speakers.

**Table 6.** Estimated Marginal Means (Probability, Diagonal) of Dark I Palatalization and Comparison Thereof for Each Speaker Group (Odds Ratio: Lower Triangle, and Significance: Upper Triangle).

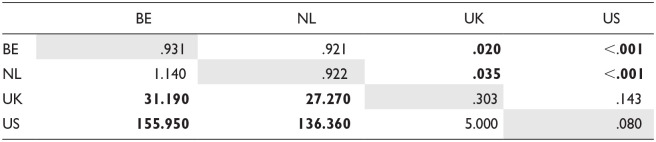

*Note*. Diagonal (gray cells): estimated marginal means (estimated probability of feature presence); lower triangle: comparison of means (odds ratio); upper triangle: significance of comparisons with FDR adjustment. Significant *p*-values (<.05) are displayed in bold.

Although most error hierarchies warn Dutch speakers against producing /l/ “over-dark” (pharyngealized; e.g., Collins & Mees, 2003; van den Doel, 2006), we did not observe any instances of [lˤ] in the transcriptions from the SAA archive. We were similarly unable to find any reference to over-extending the use of the clear *l*. Collins and Mees (2003) do mention the difference when they note that more speakers from The Netherlands should have difficulty in producing clear *l* than Belgian speakers since fewer speakers from The Netherlands have a clear *l* in their accent, but we did not see such a difference. It is also worth noting that many speakers of non-standard English accents in the United Kingdom also tend to produce clear *l* in all positions, but this was not frequent enough to yield a difference between the two groups of native English speakers here.

### 3.2 Unobserved features

Up until now we have discussed features that we observed in our data set, both new and previously described. While some of the features we found to be most distinctive of Dutch-accented English have also been included in other error hierarchies, many features commonly reported were not present among our top 25 rankings. Some of these features were in fact fairly distinctive, and could be found just beyond the top 25 items of at least some of our native-nonnative comparisons. This is the case of /w/ being realized as [v] and two UK-specific features: pronouncing “r” when it does not precede a vowel (e.g., [kɑːɹp] instead of [kɑːp] for “carp”; known as “r-insertion”) and the realization of the diphthong /əʊ/ (the GOAT vowel).

There was another set of oft-referenced features that we did not have the opportunity to observe here because the sounds or phonetic contexts eliciting them were not represented in the passage. The sounds /v/ and /z/, which are often said to be produced as [f] and [s], respectively, were an instance of this. These sounds only occurred in syllable-offset, where they were already subject to final devoicing. Other features that could not be evaluated were schwa /ə/ epenthesis between “l” and a consonant, /ʊ/ being realized as [uː], and the pronunciation of /ʒ/ and **/**dʒ/.

Finally, there were many features that could be elicited by the Stella passage but just were not found to be distinctive. In most cases this was due to the features not actually being very frequent (e.g., uvular *r*). In other instances, their absence was due to the fact that their nonnative realization patterns did not deviate enough from native English speech (e.g., /ʌ/).

For these lacking items, it is worth keeping in mind however that systematic analyses were not possible, as well as that sometimes very few—as little as one—instances were available. Further studies with more items can shed light on these features, as well as on the conditions under which they are expressed.

## 4 General discussion

The goal of this study was to demonstrate a systematic, detailed approach to study nonnative accent. To this end, we applied a method from dialectometry to a large database of phonetic transcriptions to extract the features that most distinguish the English pronunciation of Dutch speakers from Belgium and the Netherlands from that of native English speakers from the United States and United Kingdom. An overview of these features can be found in Table 2. We also demonstrated a method that can be used to verify hypotheses about accent features by checking whether the native and nonnative groups actually varied in the degree to which they expressed each feature overall. In addition, we analyzed whether there were differences between the regional varieties of the English and Dutch speakers and whether any other potential factors, such as the sound, phonetic context, or position in the syllable or word, modulated the expression of each feature. Here, we report the feature’s prevalence across the four groups of speakers and any within-feature factors for three features, as a demonstration of our approach.

With regard to the features of Dutch-accented English, our findings suggest that there is no such thing as one single Dutch accent in English but rather that it depends, in part, on the speaker’s variety of Dutch (here exemplified by differences between speakers from Belgium and The Netherlands). This finding is in line with previous work on L1 variety-specific influences on L2 perception (e.g., Escudero et al., 2012). In addition, the accent features observed here differed depending on whether the comparison was with speakers from the United States or United Kingdom, revealing that what is deemed nonnative-like varies as a function of what is considered the native standard.

Despite these caveats, there were some features that could perhaps be considered “universal” of Dutch-accented English, appearing high across our rankings. These are: word-final obstruent devoicing, dental fricative substitution, initial voiceless plosive deaspiration, /ae/ substitution, the unweakening of certain weak forms, and dark *l* palatalization. Our study thus complements previous research, where most of these features have been documented, providing empirical evidence that they are more frequently produced by Dutch speakers than native English speakers. Furthermore, we extend this work by showing that certain factors, such as the sound in question or phonetic context, may modulate their presence or specific realization. For instance, our results suggest that the two dental fricatives may be substituted to different degrees, and that Dutch speakers vary between pronouncing /æ/ as [ɛ], [a], and [æ]. This indicates that other factors may be at play during the expression of a feature during nonnative speech production, the study of which could illuminate new paths in understanding this process (see also: Munro, 2023).

While our results provide support for some known features of Dutch-accented English, they also revealed some previously undocumented features. This could be largely attributed to our bottom-up approach and the inclusion of both native and nonnative regional variability. For example, the absence of a feature regarding alveolar flapping in previous studies is likely due to a focus on RP as the native English standard. Similarly, our data suggest differences between Dutch speakers from the Netherlands and Belgium (e.g., in substitution of the voiced dental fricative). Moreover, it was thanks to both speaker variability and a more phonetically sensitive measure that we were able to observe features previously unnoticed in other bottom-up studies, such as over-extending clear *l*. Finally, the use of real speaker data instead of standards brought to light an interesting category of features distinctive not because nonnatives, but rather natives varied from the standard (e.g., /æ/ substitution among speakers from the United Kingdom). All of these findings point to the need to consider speaker variability in L2 research (Munro, 2023).

Our findings highlight the challenges of using standard pronunciations in teaching and research, rather than considering speaker variability. In particular, it is worth contemplating whether certain so-called “nonnative” features that actually coincide with certain non-standard native variety are worth correcting, especially in cases when the feature is hard to correct and/or native speakers appear so split in their realizations (e.g., the frequent realization of /æ/ as [a] seen among UK speakers). However, for this, research should further address the role of accent similarity or native familiarity with a feature in the perception of that feature or accent (e.g., see van den Doel, 2006; Wang & van Heuven, 2014). Moreover, this brings into question the value of the idea of standard accents, an issue future work should address.^2^

In addition to “new” features, there are other features commonly associated with the English pronunciation of Dutch speakers for which we did not find evidence for here. While some of these missing features were an artifact of the limited elicitation material (e.g., lack of words with [dʒ] or the cluster [lC]), others were a result of our criterion for feature selection: distinctiveness—which is a measure based on frequency—, instead of the usually-prioritized salience, intelligibility, or stigmatization (Bloem et al., 2016). The absence of the oft-cited uvular *r* and overdark *l* in our results suggest that these features may in fact not be very prevalent even if they may be salient, stereotypical, or cause misunderstandings. Yet another way in which our study varied from previous studies is that our approach is limited to the segmental and syllabic level while most studies, especially the top-down ones, include suprasegmental features among their error hierarchies (e.g., word and sentence stress, tag-question intonation; Collins & Mees, 2003; Gussenhoven & Broeders, 1997; Rupp, 2013). In addition, lack of contracted forms is often signaled (e.g., Collins & Mees, 2003; van den Doel, 2006) but cannot be assessed here because the materials were not recorded spontaneously. While we have accounted for the lack of some features, it is worth mentioning that more extensive analysis is required to understand why other features were not observed.

Thus, our results partially coincide with other error hierarchies in terms of the features observed, but our study also extends past work by analyzing additional factors that may modulate the presence of nonnative accent features, such as the sound in question (e.g., /ð/ or /θ/). Furthermore, we also provided statistical evidence of differences between natives and nonnatives, an approach which Gries (2015, 2021) has argued can enrich corpus research. Our findings thus demonstrate fruitful avenues that future work on L2 pronunciation can pursue.

More generally, our findings indicate an urgent need to bridge the gap between extensive work done in pronunciation training and experiments involving nonnative accent. As has been often noted: “Findings from linguistic research can inform teachers, and the classes at secondary schools potentially provide a wealth of data that can inform linguistic research” (van den Doel & Rupp, 2014, p. 77).

On one hand, extensive work on the perception of certain sounds (e.g., /æ/ for Dutch-accented English) has greatly expanded our knowledge of the phenomenon of L2 pronunciation. Nonetheless, overviews of accent features like ours are a reminder that much remains to be understood about the different types of processes involved in nonnative accent. To illustrate, take our list, with features ranging from the replacement of novel L2 sounds (e.g., /æ/, /ð/), to the misapplication of phonological rules that exist in the L1 (e.g., the use of weak forms or clear vs. dark *l*). To understand processes such as nonnative speech production or the perception of nonnative accent, not to mention the large individual differences in the acquisition of nonnative phonology, a comprehensive perspective of accent is necessary. By studying less-documented features we may be able to open a new window into the phenomenon of accent.

On the other hand, impressionistic work could use quantitative support to discover patterns that may be subject to bias or easily escape immediate observation (e.g., sound differences, or features only appearing under certain conditions). Our work demonstrates that bottom-up work can provide empirical support for these features, as well as yield more information. Moreover, it can also indicate the prevalence of features or even introduce new ones. Information about the frequency of features and the conditions modulating their expression can also guide researchers in selecting features to study. In this way, our results add to the collection of tools available for English teachers and researchers of Dutch-accented English, in addition, allowing them to focus on the features specific to Dutch speakers from Belgium or The Netherlands, as well as relative to American or British English pronunciation.

In 2016, Bloem and collaborators proposed the use of aggregate phonetic distances with transcriptions to study nonnative accents, but, to our knowledge, the present study is the first to try this. We hope that after this demonstration more studies make use of this approach to study other accents, especially less-documented ones, where extensive overviews of features may be lacking. In this regard it is worth noting that the SAA has hundreds of speech samples of speakers of many different languages or dialects annotated and ready to be analyzed, although unfortunately only of English. Researchers interested in applying our approach are limited by the availability of transcriptions, which involve their own bias, as well as the time-consuming steps of alignment and segmentation. However, these could be facilitated in the future by using automatic speech recognition technology (e.g., Xie et al., 2018).

Another limitation of our approach is that the phonetic distance measure implemented in Gabmap is still fairly broad (e.g., [b-b̥] has the same distance as [b-v̥]). However, the web application does have a feature allowing you to define your own string edit distances. Moreover, since the time this study was conducted, phonetic features (i.e., attributes such as place of articulation and voicing) have been implemented to better gauge phonetic distance (see, e.g., the updated LED-A; Heeringa et al., 2022). In the future, perceptual or acoustic research could also be used to inform these edit distances.

In addition to these limitations, as already mentioned, the features one is able to observe with this approach are limited by the elicitation material. In this regard, it is difficult to guarantee the presence of all sounds in all contexts, with enough instances of each to allow analysis. Moreover, one advantage of the Stella passage is precisely its reduced length and we maintain the usefulness of material such as the Stella passage in providing an indication of potential features to be further investigated. Nonetheless, future work could be aimed at developing more exhaustive elicitation material, perhaps specifically oriented toward specific accents. In addition to the elicitation material, the results of our study are also limited to the (unbalanced) samples available in the corpus, which sometimes disproportionately represented certain regions of the countries concerned.

Besides remediating these limitations, another obvious follow-up would be to include English samples from other nonnative speakers to see how Dutch speakers’ pronunciation varies from that of other nonnative speakers (e.g., German). This would be informative for research into accent detection and identification as it would not only show how Dutch speakers vary from native English speakers, but also from native speakers of other languages. Finally, future research should also investigate how different features specifically impact detection, intelligibility, and stigmatization.

## 5 Conclusion

In this study, we outlined a systematic approach to study the features of nonnative pronunciation based on speaker samples. Here, we demonstrated this approach by describing its application to determine the most distinctive features of Dutch-accented English, but it can be used to study any nonnative, not to mention native, accent. Our findings were in part consistent with previous work on Dutch-accented English, but revealed some new insights thanks to the use of a data-driven approach, the inclusion of speaker and regional variability among both the natives and nonnatives, the use of a phonetically sensitive and frequency-based difference measure, and the analysis of factors potentially modulating feature expression. We hope to have demonstrated the value of this approach in particular and quantitative approaches in general for the study of nonnative accent features. Furthermore, our study casts a light on promising areas for future research on nonnative pronunciation. It is our hope that this study and the results thereof can be useful to pronunciation teachers and researchers alike.

## Appendix A

Segmentation of Stella Passage Into “Items” for Analysis.

## Appendix B

### Potential items where each feature could have occurred

#### Dental fricative substitution

Please call Stella. Ask her to bring [th]ese [th]ings wi[th] her from [th]e store: Six spoons of fresh snow peas, five [th]ick slabs of blue cheese, and maybe a snack for her bro[th]er Bob. We also need a small plastic snake and a big toy frog for [th]e kids. She can scoop [th]ese [th]ings into [th]ree red bags, and we will go meet her Wednesday at [th]e train station.

#### /æ/ substitution

Please call Stella. [A]sk her to bring these things with her from the store: Six spoons of fresh snow peas, five thick sl[a]bs of blue cheese, [a]nd maybe a sn[a]ck for her brother Bob. We also need a small pl[a]stic snake [a]nd a big toy frog for the kids. She c[a]n scoop these things into three red b[a]gs, [a]nd we will go meet her Wednesday [a]t the train station.

#### Dark *l* palatalization

P[l]ease ca[ll] Ste[ll]a. Ask her to bring these things with her from the store: Six spoons of fresh snow peas, five thick s[l]abs of b[l]ue cheese, and maybe a snack for her brother Bob. We a[l]so need a sma[ll] p[l]astic snake and a big toy frog for the kids. She can scoop these things into three red bags, and we wi[ll] go meet her Wednesday at the train station.

## Appendix C

### Rankings of most distinctive items for the four speaker group comparisons

**Table C1.** Top 25 Items in US-NL Comparison.

Rank	Distinctiveness score	Item		Most frequent realizations^a^		Feature
		#	in context	US	NL	
1	1.64	76	slabs	Z	s	word-final obstruent devoicing
2	1.62	75	slabs	B	p/b̥	word-final obstruent devoicing
3	1.46	140	big	ɡ/ɡ̥̚	k	word-final obstruent devoicing
4	1.40	149	the [2]	Ð	d	dental fricative substitution
5	1.23	53	spoons	Z	s/z	word-final obstruent devoicing
6	1.20	183	bags	ɡ	ɡ̥/k	word-final obstruent devoicing
7	1.14	13	ask	Æ	a/æ	/æ/ substitution
8	1.11	65	peas	z/z̥	s	word-final obstruent devoicing
9	1.06	184	bags	z/z̥	s	word-final obstruent devoicing
10	1.03	1	please	pʰ	p	initial voiceless plosive deaspiration
11	0.99	84	cheese	Z	s/z/z̥	word-final obstruent devoicing
12	0.93	170	things [2]	Z	z̥/s	word-final obstruent devoicing
13	0.89	146	frog	ɡ	k/ɡ̥/ɡ	word-final obstruent devoicing
14	0.88	108	Bob	B	p/b̥	word-final obstruent devoicing
15	0.87	68	five	V	f	word-final obstruent devoicing
16	0.86	4	please	z/z̥	s	word-final obstruent devoicing
17	0.82	164	these [2]	Ð	ð/d̪/d	dental fricative substitution
18	0.79	104	brother	Ð	ð	dental fricative substitution
19	0.76	30	things [1]	Z	s/z	word-final obstruent devoicing
20	0.73	153	kids	D	t/d̥/d	word-final obstruent devoicing
21	0.73	208	the [3]	Ð	d̪/ð	dental fricative substitution
22	0.71	95	snack	Æ	æ	/æ/ substitution
23	0.68	18	to	ɾ	t/tʰ	alveolar stop unflapping
24	0.64	163	scoop	P	p/p̬	regressive voice assimilation
25	0.62	26	these [1]	z/z̥	s/z̥	word-final obstruent devoicing

Note: Words that occur multiple times in the paragraph have a number next to them to indicate the instances being referred to.

a Realizations with relative frequencies ⩾ 25%.

**Table C2.** Top 25 Items in UK-NL Comparison.

Rank	Distinctiveness score	Item		Most frequent realizations^a^		Feature
		#	in context	UK	NL	
1	0.61	76	slabs	Z	s	word-final obstruent devoicing
2	0.59	149	the [2]	Ð	d	dental fricative substitution
3	0.58	1	please	pʰ	p	initial voiceless plosive deaspiration
4	0.57	68	five	v/v̥	f	word-final obstruent devoicing
5	0.53	65	peas	z/z̥	s	word-final obstruent devoicing
6	0.52	174	into	ə	u	weak form unweakening
7	0.51	140	big	ɡ/ɡ̥	k	word-final obstruent devoicing
8	0.45	123	plastic	pʰ	p	initial voiceless plosive deaspiration
9	0.44	75	slabs	B	p/b̥	word-final obstruent devoicing
10	0.39	170	things [2]	Z	z̥/s	word-final obstruent devoicing
11	0.37	53	spoons	Z	s/z	word-final obstruent devoicing
12	0.37	148	for [2]	ə	ɚɹ	weak form unweakening
13	0.35	4	please	z/z̥	s	word-final obstruent devoicing
14	0.35	84	cheese	Z	s/z/z̥	word-final obstruent devoicing
15	0.35	30	things [1]	Z	s/z	word-final obstruent devoicing
16	0.35	98	for [1]	ə/ʰ	ʰɹ	weak form unweakening
17	0.30	164	these [2]	Ð	ð/d̪/d	dental fricative substitution
18	0.30	184	bags	z/z̥	s	word-final obstruent devoicing
19	0.29	40	the [1]	ð/n̪	d̪/n̪	dental fricative substitution
20	0.29	208	the [3]	Ð	d̪/ð	dental fricative substitution
21	0.27	108	Bob	b/b̥	p/b̥	word-final obstruent devoicing
22	0.26	183	bags	ɡ	ɡ̥/k	word-final obstruent devoicing
23	0.26	5	call	kʰ/k	k	initial voiceless plosive deaspiration
24	0.26	166	these [2]	Z	s	word-final obstruent devoicing
25	0.25	19	to	ə	u/ə	weak form unweakening

Note. Words that occur multiple times in the paragraph have a number next to them to indicate the instances being referred to.

a Realizations with relative frequencies ⩾ 25%.

**Table C3.** Top 25 Items in US-BE Comparison.

Rank	Distinctiveness score	Item		Most frequent realizations^a^		Feature
		#	in context	US	BE	
1	1.18	140	big	ɡ/ɡ̥̚	k	word-final obstruent devoicing
2	0.99	75	slabs	B	b/p/b̥	word-final obstruent devoicing
3	0.98	76	slabs	Z	s	word-final obstruent devoicing
4	0.95	13	ask	Æ	a	/æ/ substitution
5	0.95	53	spoons	Z	s/z̥	word-final obstruent devoicing
6	0.88	84	cheese	Z	s/z	word-final obstruent devoicing
7	0.78	4	please	z/z̥	s	word-final obstruent devoicing
8	0.78	149	the [2]	Ð	ð/d̪	dental fricative substitution
9	0.74	184	bags	z/z̥	s/z̥	word-final obstruent devoicing
10	0.73	65	peas	z/z̥	s	word-final obstruent devoicing
11	0.71	170	things [2]	Z	s/z̥/z	word-final obstruent devoicing
12	0.69	30	things [1]	Z	s/z̥	word-final obstruent devoicing
13	0.69	183	bags	ɡ	ɡ̥/ɡ	word-final obstruent devoicing
14	0.65	146	frog	ɡ	ɡ̥/ɡ	word-final obstruent devoicing
15	0.57	208	the [3]	Ð	d̪/ð	dental fricative substitution
16	0.55	18	to	ɾ	t	alveolar stop unflapping
17	0.47	70	thick	ɪ	ɪ	/ɪ-i/ conflation
18	0.46	153	kids	D	d/t/d̥	word-final obstruent devoicing
19	0.44	1	please	pʰ	pʰ/p	initial voiceless plosive deaspiration
20	0.43	108	Bob	B	b̥/b	word-final obstruent devoicing
21	0.43	166	these [2]	Z	s/z̥	word-final obstruent devoicing
22	0.41	175	three	Θ	θ	dental fricative substitution
23	0.40	73	slabs	L	l/l̥	approximant devoicing
24	0.40	33	with	Θ	θ	dental fricative substitution
25	0.40	21	bring	ɹ	ɹ	alveolar trill *r*

Note. Words that occur multiple times in the paragraph have a number next to them to indicate the instances being referred to.

a Realizations with relative frequencies ⩾ 25%.

**Table C4.** Top 25 Items in UK-BE Comparison.

Rank	Distinctiveness score	Item		Most frequent realizations^a^		Feature
		#	in context	UK	BE	
1	0.45	16	her [1]	∅	h	weak form unweakening
2	0.44	140	big	ɡ/ɡ̥	k	word-final obstruent devoicing
3	0.39	4	please	z/z̥	s	word-final obstruent devoicing
4	0.32	84	cheese	Z	s/z	word-final obstruent devoicing
5	0.31	7	call	lˠ/l	l	dark *l* palatalization
6	0.31	123	plastic	pʰ	p	initial voiceless plosive deaspiration
7	0.28	30	things [1]	Z	s/z̥	word-final obstruent devoicing
8	0.27	65	peas	z/z̥	s	word-final obstruent devoicing
9	0.22	166	these [2]	Z	s/z̥	word-final obstruent devoicing
10	0.21	170	things [2]	Z	s/z̥/z	word-final obstruent devoicing
11	0.21	76	slabs	Z	s	word-final obstruent devoicing
12	0.21	174	into	ə	u	weak form unweakening
13	0.21	53	spoons	Z	s/z̥	word-final obstruent devoicing
14	0.20	148	for [2]	ə	ɚ	weak form unweakening
15	0.17	149	the [2]	Ð	ð/d̪	dentral fricative substitution
16	0.16	184	bags	z/z̥	s/z̥	word-final obstruent devoicing
17	0.15	208	the [3]	Ð	d̪/ð	dentral fricative substitution
18	0.15	89	maybe	eɪ	eɪ	realization of /eɪ/
19	0.13	98	for [1]	ə/ɚ	ɚ/ɚɹ	weak form unweakening
20	0.13	182	bags		Æ	/æ/ substitution
21	0.12	205	Wednesday	eɪ	eɪ	realization of /eɪ/
22	0.12	75	slabs	B	b/p/b̥	word-final obstruent devoicing
23	0.11	74	slabs	Æ	Æ	/æ/ substitution
24	0.11	107	Bob		ɑ	open back vowel unrounding
25	0.10	64	peas	iː	iː	/iː/ monophthongization

Note 1. Words that occur multiple times in the paragraph have a number next to them to indicate the instances being referred to.

Note 2. “∅” indicates that the sound was omitted altogether. An empty cell indicates that none of the realizations had a relative frequency ⩾ 25% for that item.

a Realizations with relative frequencies ⩾ 25%.

## Appendix D

### Mean relative frequency of feature per speaker group and, if applicable, condition

**Table:** Dental fricative substitution.

	Speaker group			
Sound	BE	NL	UK	US
/ð/	37.16	60.42	4.65	4.69
/θ/	32.00	18.75	16.51	10.08

*Note*. Values in percentages.

**Table:** /æ/ substitution.

Speaker group			
BE	NL	UK	US
56.00	66.25	76.15	50.50

*Note*. Values in percentages.

**Table:** Dark *l* palatalization.

	Speaker group			
Sound	BE	NL	UK	US
/l/	100.00	100.00	98.18	98.18
/ɫ/	73.47	73.02	43.53	29.92

*Note*. Values in percentages.

## Appendix E

### Estimated marginal means (probability, diagonal) of each feature per condition and comparison thereof for each speaker group (odds ratio: lower triangle, and significance: upper triangle)

**Table 4:** (from section 3.1.1 Dental fricative substitution) C-F:.

**C**	BE		**D**	NL	
	ð	θ		ð	θ
ð	.329	.685	ð	.645	.001
θ	0.721	.261	θ	0.082	.130
	UK			US	
E	ð	θ	F	ð	θ
ð	0.014	.031	ð	0.016	.050
θ	8.467	0.109	θ	3.808	0.059

## Notes

1. Realization of a dental fricative as a nasal due to assimilation was not counted toward this feature.

2. Thanks to an anonymous reviewer for highlighting this.
